# Litigation following traumatic brain injury—what are the challenges and what can we do?

**DOI:** 10.1093/intqhc/mzaf107

**Published:** 2025-10-07

**Authors:** Nichola Robson, Gemma Bradley, Rebecca Morris, Lee Jones, Zoe Jones

**Affiliations:** Community Acquired Brain Injury Service, Cumbria, Northumberland, Tyne and Wear NHS Foundation Trust, Gateshead, Sunderland and South Tyneside, UK; School of Communities and Education, Northumbria University, Coach Lane Campus, Newcastle Upon Tyne, Tyne and Wear, NE7 7XA, UK; Community Acquired Brain Injury Service, Cumbria, Northumberland, Tyne and Wear NHS Foundation Trust, Gateshead, Sunderland and South Tyneside, UK; Community Acquired Brain Injury Service, Patient and Public Involvement Contributor. Gateshead, Sunderland and South Tyneside, UK; Community Acquired Brain Injury Service, Patient and Public Involvement Contributor. Gateshead, Sunderland and South Tyneside, UK

## Background to a Current and Evolving Problem 

Traumatic Brain Injury (TBI) is a major global public health concern. In 2019, there were estimated to be over 27million new cases of TBI worldwide, and 7.08million years lost to disability [^1^]. Common causes of TBI include falls, road traffic accidents, and violence [^2^] with a significant proportion (approximately 41%) resulting in litigation [^3^]. Global incidence is likely to continue to increase due to factors such as population density, ageing and increasing use of vehicles [^4^] and it is therefore reasonable to predict numbers of litigation cases will also rise.

Litigation is a prolonged, often adversarial process which has been shown to exacerbate TBI symptoms and link to poorer outcomes including likelihood of depression and difficulties with return to work [^5^]. Poorer outcomes for those going through litigation are also reported across other populations. A recent systematic review exploring the effects of litigation after all types of traumatic injury found a positive trend towards poorer psychological, somatic, disability and quality of life outcomes [^6^].

There are complex ethical dilemmas for healthcare professionals working with survivors of TBI as they go through a litigation journey. Firstly, the awareness of poorer outcomes for people who embark on litigation raises issues of information-sharing and supporting informed decisions. Also, depending on the healthcare system, professionals may be asked to share evaluations or case notes that are used in litigation proceedings, at the same time as providing person-centred rehabilitation and support. Competing values to improve someone’s situation, avoid harm and yet meet employer or contractual requirements can raise significant tensions.

There is significant debate surrounding the enhanced symptomatology and reported poorer outcomes. One dominant narrative is linked to suboptimal effort, symptom exaggeration and malingering motivated by secondary, often financial, gain [^7^]. The term *compensation neurosis* was used as far back as 1961 [^8^] to describe non-objectifiable symptoms which reportedly resolve following litigation settlement. Another explanation, mainly drawing inferences from populations with musculoskeletal injury, is that individuals with more or worse symptoms are more likely to initiate litigation [^9^]. However another is that litigation itself can negatively influence reported symptoms and health outcomes, attributed to factors such as additional stress, cognitive load and disruption to rehabilitation. In reality, enhanced symptomology is likely to be individual and influenced by multiple factors although we primarily build on this third example to examine how we can reduce stress through integrated, empathetic and responsive services.

Authors of this *Perspectives* paper include a person and a family member who have lived through litigation following TBI (LJ, ZJ). Their words are indicated through *italicized* extracts. Co-authors include health professionals from neuropsychology (NR, RM) and occupational therapy (GB) working in community acquired brain injury rehabilitation in the UK. We firstly aim to develop further understanding of the above problem by drawing on the perspectives of those who have directly experienced it. We then draw on this experience to suggest starting points for survivors, families, health and legal professionals to work towards shared understanding and improved services. Finally, we emphasise that additional challenges should not be inevitable for survivors of TBI who are involved in litigation, and we begin a call to action for global developments in research and practice.

## Characterizing the Problem from the Perspective of those with Lived Experience

“Our experience is unique and often invisible—how can we make people understand?”

TBI is often referred to as a hidden disability with 76% of survivors experiencing daily problems as a direct consequence of their injury being hidden [^10^]. TBI symptoms can be difficult to explain; further complicated when cognition and communication skills are impaired. Yet during litigation, people are required to explain and justify symptoms and impairments in ways which can be observed and measured. This can contribute to feeling misunderstood and “*like a fraud*.*”* Potentially damaging narratives emerge which can exacerbate these feelings; for example, that individuals develop a sense of entitlement to financial compensation, or that symptom exaggeration is commonplace. The process can be overwhelming, distressing, confusing, and *“makes you constantly question yourself*.*”*
 

“No closure until settlement”

*“Litigation is all-consuming, it invades every aspect of your life. It makes you feel untrustworthy, paranoid, confused, and lost*.*”* There is significant cognitive and emotional load associated with litigation; re-living traumatic experiences, attending meetings and appointments, and managing large amounts of complex financial, medical, and legal information. This is in addition to engaging with demanding and unpredictable rehabilitation and adjusting to significant, often lifelong changes, whilst having depleted physical, cognitive, and psychological resources.

Litigation can last up to eight years [^3^] yet access to professional, multi-agency support during this time is likely to be highly variable. Whilst dealing with the additional cognitive load, survivors and family members often face immediate financial pressures and longer-term uncertainty. It is sometimes only after settlement that survivors *“relearn how to live a life with brain injury”* and truly begin the adjustment process.

## Priority Areas for Individuals, Practitioners and Services

### Recommendations for individuals and families

Symptoms can feel worse when also feeling frustration, helplessness or when experiences are called into question. Therefore, maintaining a sense of control could help. Examples include keeping diaries of symptoms, records of time needed for carer support, and receipts of expenditure. Taking someone to appointments can also be beneficial to support recall and provide advocacy.

Recommendations for healthcare professionals

Information and support for the individual and their family about stages of the litigation process is essential. Services can also facilitate peer-support opportunities for survivors and caregivers to share and explore their experiences.

Education for health and legal professionals working with survivors of TBI is also imperative. We suggest developing co-designed induction and training resources for professionals, alongside accessible information in different formats. People also value organisations working together whilst keeping them at the centre of decision-making. Furthermore, such partnerships can become learning communities, raising awareness and bringing together like-minded people who innovate for change.

Recommendations for organisations and policymakers

Community rehabilitation is sometimes episodic and time-limited, which may mean support is not available at important times. We recommend avoiding reductive practices (such as discharging people who miss appointments) and considering ways to engage or re-engage with support throughout the litigation journey.

We also suggest inter-agency working to draw upon legal, financial, psychological and practical support at times when they are needed. This can help to reduce inherent risks to individuals during the litigation process. We call on policymakers to explore innovative models for this within their own healthcare contexts.

A further challenge is to develop and embed assessment and measurement tools throughout litigation which are sufficiently sensitive to evaluate changes to the lives of TBI survivors and their families. We advocate for a biopsychosocial approach which includes objective and subjective measures.

## Conclusions

We hope our coproduced paper raises awareness of the experience of litigation following TBI and highlights the complex issues faced by survivors and family members. Whilst we have proposed actions for individuals, professionals, organisations and policy makers, there are also areas which need focussed and collaborative work.

We suggest further research is needed. Studies to further understand lived experience amongst different groups and within different contexts is important, particularly where different medical and legal systems are likely to be significant. We have also been unable to find specific examples of integrated rehabilitation and litigation support and there is an opportunity to develop and evaluate ways of working which provide early and targeted help and promote coping and self-management throughout the process.

## Data Availability

No new data were generated or analysed in support of this manuscript.
